# Laboratory-based surveillance of *Shigella* spp. from human clinical cases in Colombia, 1997-2018

**DOI:** 10.7705/biomedica.5113

**Published:** 2020-09-09

**Authors:** Edna Catering Rodríguez, Adriana Marcela Bautista, Lucy Angeline Montaño, María Victoria Ovalle, Francia Patricia Correa

**Affiliations:** Grupo de Microbiología, Dirección de Redes en Salud Pública, Instituto Nacional de Salud, Bogotá, D.C., Colombia

**Keywords:** Dysentery, bacillary, public health surveillance, drug resistance, microbial, ampicillin, cephalosporins, fluoroquinolones, trimethoprim, sulfamethoxazole drug combination, chloramphenicol, disentería bacilar, vigilancia en salud pública, farmacorresistencia microbiana, ampicilina, cefalosporinas, fluoroquinolonas, combinación trimetoprim y sulfametoxazol, cloranfenicol

## Abstract

**Introduction:**

Shigellosis is endemic in low-and middle-income countries, causing approximately 125 million episodes of diarrhea and leading to approximately 160 .000 deaths annually one-third of which is associated with children.

**Objective:**

To describe the characteristics and antimicrobial resistance profiles of *Shigella* species recovered in Colombia from 1997 to 2018.

**Materials and methods:**

We received isolates from laboratories in 29 Colombian departments. We serotyped with specific antiserum and determined antimicrobial resistance and minimal inhibitory concentrations for ten antibiotics with Kirby-Bauer tests following the Clinical and Laboratory Standards Institute recommendations.

**Results:**

We analyzed 5,251 isolates of *Shigella* spp., most of them obtained from stools (96.4%); 2,511 (47.8%) were from children under five years of age. The two most common species were *S. sonnei* (55.1%) and *S. flexneri* (41.7%). The highest resistance rate was that of tetracycline (88.1%) followed by trimethoprim-sulfamethoxazole (79.3%) and ampicillin (65.5%); 50.8% of isolates were resistant to chloramphenicol, 43.6% to amoxicillin/clavulanic acid, and less than 1% to cefotaxime, ceftazidime, gentamicin, and ciprofloxacin. In *S. sonnei*, the most common resistance profile corresponded to trimethoprim-sulfamethoxazole (92%) whereas in *S. flexneri* the most common antibiotic profiles were multidrug resistance.

**Conclusions:**

In Colombia, children under five years are affected by all *Shigella* species. These findings should guide funders and public health officials to make evidence-based decisions for protection and prevention measures. The antimicrobial resistance characteristics found in this study underline the importance of combating the dissemination of the most frequently isolated species, *S. sonnei* and *S. flexneri*.

## Abstract (es)

**Introducción:**

La shigelosis es endémica en los países de ingresos bajos y medios y ocasiona aproximadamente 125 millones de episodios de diarrea y 160.000 muertes al año, un tercio de los cuales se presenta en niños.

**Objetivo:**

Describir las características y los perfiles de resistencia antimicrobiana en aislamientos de *Shigella* spp. recuperados en Colombia entre 1997 y 2018.

**Materiales y métodos:**

Los aislamientos provenían de laboratorios en 29 departamentos de Colombia. La serotipificación se hizo con antisueros específicos de *Shigella* spp. y, la determinación de los perfiles de resistencia y la concentración inhibitoria mínima de diez antibióticos, por Kirby-Bauer.

**Resultados:**

Se estudiaron 5.251 aislamientos de *Shigella* spp. obtenidos de materia fecal (96,4%); el 47,8% de ellos correspondía a niños menores de cinco años. Las especies más frecuentes fueron *S. sonnei* (55,1%) y *S. flexneri* (41,7%). Se presentó resistencia a tetraciclina (88,1%), trimetoprim-sulfametoxasol (79,3%), ampicilina (65,5%), cloranfenicol (50,8%) y amoxicilina-acido clavulánico (43,6%). La resistencia no superó el 1% contra cefotaxime, ceftazidima, gentamicina y ciprofloxacina. Para *S. sonnei*, el perfil de resistencia más frecuente correspondió a trimetoprim-sulfametoxasol, en contraste con *S. flexneri,* cuyos perfiles fueron todos multirresistentes.

**Conclusiones:**

Los niños menores de cinco años se vieron afectados por todas las especies de *Shigella* spp., aspecto que los legisladores en salud pública deben considerar a la hora de tomar decisiones en torno a las medidas de prevención y protección frente a esta enfermedad. Las características de resistencia antimicrobiana de los aislamientos de *Shigella* spp. en Colombia ponen de manifiesto la importancia de combatir la diseminación de las dos especies más frecuentes en casos clínicos, *S. sonnei* y *S. flexneri*.

Edna Catering Rodríguez, Grupo de Microbiología, Dirección de Redes en Salud Pública, Instituto Nacional de Salud, Avenida Calle 26 N° 51-20 CAN, Bogotá, D.C., Colombia

Edna Catering Rodríguez: manuscript conception, design, and writing

All authors participated in the data analysis and interpretation, the critical review of important intellectual content, and the approval of the final version, and they assume full responsibility for all aspects of the manuscript.

Laboratory Surveillance on *Shigella* spp. by the *Instituto Nacional de Salud* microbiology laboratory has received support from Colciencias through the project "Fortalecimiento de la capacidad diagnóstica de investigación y de vigilancia de enfermedades transmisibles emergentes y reemergentes en Colombia".

Diarrhea is a major global health issue causing 1.3 million deaths each year 500.000 of which occur in children less than five years of age (1). Shigellosis is endemic in most low- and middle-income countries and is the most important cause of bloody diarrhea worldwide (2). Recent estimates attribute to *Shigella* spp. approximately 125 million diarrhea episodes annually leading to approximately 160,000 deaths one-third of which are associated with young children (1,3). The Global Enteric Multicenter Study revealed that *Shigella* spp. was the most prevalent causative agent in children aged 2 to 5 years who experienced diarrhea and suggested that the induced burden may be twice as high as previously estimated (4).

*Shigella* genus is divided into the following four species and into multiple serotypes dependent on O-antigen and biochemical differences: *Shigella dysenteriae* (serogroup A, 15 serotypes), *Shigella flexneri* (serogroup B, 19 serotypes), *Shigella boydii* (serogroup C, 20 serotypes), and *Shigella sonnei* (serogroup D, 1 serotype) (5). The pathogen spreads by direct contact with an infected person or by ingesting contaminated food or water. The infective dose can be as low as 10 microbes; its global importance arises from its wide distribution and water quality concerns that make it an important risk for public health (6). *Shigella sonnei* and *S. boydii* usually cause relatively mild illness with watery or bloody diarrhea while *S. flexneri* is the chief cause of endemic shigellosis in low- and middle-income countries (7).

Many observations have concluded that *Shigella* species are geographically stratified based on the level of economic development in each country. *Shigella flexneri* is the primary infectious species in low- and middle-income countries and *S. sonnei* rates increase with economic development. *Shigella boydii* is usually restricted to Bangladesh and South-East Asia rarely occurring outside these regions while *S. dysenteriae* type 1 (Sd1) outbreaks occur sporadically (8). Emerging multidrug-resistant *Shigella* isolates have exacerbated the public health impact of shigellosis leading to increased morbidity, mortality, and treatment costs (2,9).

Given that *Shigella* is a major contributor to the global diarrhea burden, vaccination can be an effective strategy to prevent the disease. However, there are at least 50 recognized *Shigella* serotypes and their distribution differs among geographical regions hindering vaccine development (10).

Public health significant microorganisms in Colombia include *Shigella* spp. and *Salmonella* spp. These species are part of the surveillance of acute diarrheal diseases by public health laboratories and the *Laboratorio de Microbiología* at *Instituto Nacional de Salud*. Given the need to better understand the behavior of *Shigella* species at a national level, we report here the age of the population affected, the temporal and geographical characteristics of the isolates, and the antimicrobial resistance profiles of *Shigella* species recovered in the country from 1997 to 2018.

## Materials and methods

Strains recovered from patients were collected in hospitals or public health laboratories and then sent to the *Laboratorio de Microbiología* at the *Instituto Nacional de Salud* to confirm their biochemical identification following the standardized procedures and to serotype them using commercial polyclonal and monoclonal typing antisera (Eurobium) in the framework of the acute diarrheal disease surveillance program (11).

Antimicrobial resistance was determined using the Kirby-Bauer test against tetracycline (30 μg), chloramphenicol (30 μg), nalidixic acid (30 μg), amoxicillin/clavulanic acid (10 μg), and ciprofloxacin (5 μg). Additionally, we obtained the minimal inhibitory concentrations (MIC) for ampicillin (8-16 μg/ml), cefotaxime (2-32 μg/ml), ceftazidime (1-16 μg/ml), ciprofloxacin (1-2 μg/ml), gentamicin (4-86 μg/ml), and trimethoprim-sulfamethoxazole (2/38 μg/ml) with the Microscan® (Siemens) automated system following the recommendations of the Clinical and Laboratory Standards Institute yearly updated (12). Statistical significance (p value) was calculated with EpiInfo 7.

## Results

We studied 5,251 isolates of *Shigella* spp. recovered in 29 Colombian departments. Bogotá, the country’s capital city, sent the highest number of isolates (n=3,257, 62%). Another five states recovered more than one hundred isolates each: Antioquia (n=814; 15%); Valle (n=205; 3.9%); Norte de Santander (n=187; 3.6%); Nariño (n=157; 3%), and Boyacá (n=146; 2.8%). The additional 485 isolates were recovered from the other 23 departments.

The majority of isolates were obtained mainly from stools (96.4%; 5,063/5,251) of patients whose ages ranged from newborns to 92 years. The children under one year of age contributed 452 isolates (8.6%), and the patients between 2 and 5 years contributed 2,059 isolates (39.2%); 1,498 (28.5%) isolates were recovered from 6 to 14-year-old patients while 904 isolates (17.2%) were recovered from those over 15 years (Table 1).

**Table 1:** Age and sample distribution

	**Age in years**					
**Sample type**	**<1**	**2- 5**	**6 - 14**	**>15**	**ND**	**Total**
Stool	435	2,012	1,469	834	313	5,063
NC	6	24	15	18	18	81
Blood	8	10	4	36	6	64
Urine	2	11	4	13		30
Other	1	2	6	3	1	13
Total	452	2,059	1,498	904	338	5,251

NC: Information not collected

Two species were predominant representing together 96% of the strains: 2,896 (55.1%) isolates were *S. sonnei* and 2,191 (41.7%) were *S. flexneri*. Other species included *S*. *boydii* and *S*. *dysenteriae* with 103 and 14 isolates each. Some of the isolates had some of the typical characteristics of *Shigella* but it was not possible to identify them at the species level and, therefore, they were labeled as non-serotypeable *Shigella*. Non-serotypeable *Shigella* accounted for approximately 47 isolates, none of which were agglutinated with any antisera of the established *Shigella* serovars.

**Figure 1:**
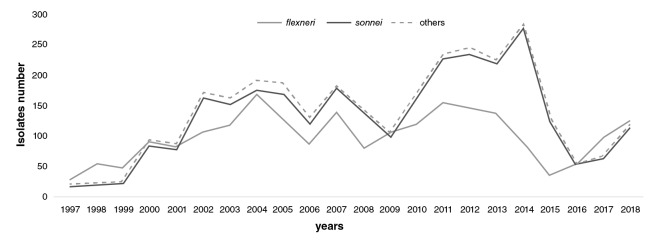
Distribution of *Shigella* spp. isolates in Colombia from 1997 to 2018. *S. flexneri* (grey), *S. sonnei* (black), and other species (dashed line). This information was based on the annual *Shigella* surveillance system in Colombia.

During the first three years of surveillance (1997-1999), *S. flexneri* was the dominant species decreasing after 2002 when *S. sonnei* became the most dominant and continued to be for 15 years, until 2016, when it became again the second most common strain (figure 1).

The highest resistance level was found for tetracycline (88.1%) followed by trimethoprim-sulfamethoxazole (79.3%), and ampicillin (65.5%); 50.8% of isolates were resistant to chloramphenicol and 43.6% to amoxicillin/clavulanic acid. Less than 1% of the isolates were resistant to cephalosporins (cefotaxime and ceftazidime), gentamicin, and ciprofloxacin (table 2).

**Table 2:** Antimicrobial resistant isolates distribution in different *Shigella* species

***Shigella* species**	**Antibiotics**									
	**AMC**	**TE**	**C**	**NA**	**CAZ**	**CTX**	**AMP**	**SXT**	**CIP**	**GM**
*boydii*	12.3	83.5	10.7	3.13	2.47	0.97	76.7	51.5	0	1.06
*dysenteriae*	0	41.7	7.14	11.1	0	0	28.6	42.9	0	0
*flexneri*	60.7	94.3	80.8	2.34	0.22	0.37	83	67.8	0.41	0.27
*Shigella* spp.	12.5	63.8	27.7	9.76	0	0	61.4	65.9	2.17	0
*sonnei*	32.8	83.9	29.4	7.15	0.4	0.43	52	89.6	0.49	0.37
Total	43.6	88.1	50.8	5.13	0.36	0.41	65.5	79.3	0.46	0.34

AMC: Amoxicillin/clavulanic acid; TE: Tetracycline; C: Chloramphenicol; NA: Nalidixic acid; CAZ: Ceftazidime; CTX: Cefotaxime; AMP: Ampicillin; SXT: Trimethoprim-sulfamethoxasole; CIP: Ciprofloxacin; GM: Gentamicin

### Geographical distribution, age, and antimicrobial resistance of species

#### Shigella sonnei

**Figure 2:**
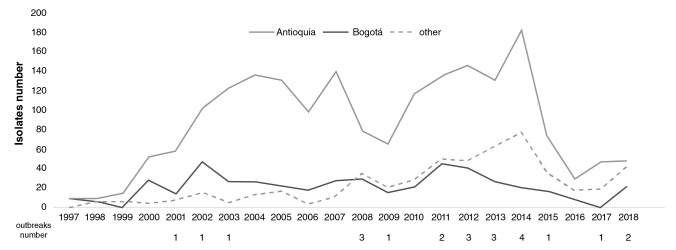
Comparison of the number and distribution of *Shigella sonnei* isolates in Antioquia (black), Bogotá (grey), and other regions (dashed line) in Colombia from 1997 to 2018

Geographical distribution: *S. sonnei* represented more than half of the Colombian *Shigella* spp. isolates evaluated (2,896, 54.7%); 66% (1,975/2,896) of the isolates under study were recovered in Bogotá: 58 isolates detected in 2001 and 101 in 2002; 16% of isolates were recovered in Antioquia while the remaining 17.8% were from the other 25 departments where the recovery increased between 2011 and 2014 as the highest number of isolates for this species came from those states in the last few years (figure 2).

There were 23 outbreaks in nine different departments (Antioquia, Bogotá, Boyacá, Cundinamarca, Nariño, Norte de Santander, Santander, Sucre, and Valle) with 80 related isolates. The outbreak that grouped most cases (12) occurred in Nariño in 2008. The isolates recovered in the other outbreaks registered there varied.

Age: Almost half of the isolates (48%, 1390/2896) were recovered from children under 6 years of age. In the 6 to 14-year age group, *S. sonnei* was the most frequently recovered (32.6% of isolates; 943/2896) (table 3).

**Table 3:** Age range and *Shigella* species distribution

	***Shigella* species**					
**Age in years**	***boydii***	***dysenteriae***	***flexneri***	***Shigella* spp.**	***sonnei***	**Total**
<1	6.8	0.0	11.2	9.9	6.7	8.6
2 to 5	30.1	28.6	37.2	34.1	41.3	39.3
6 to 14	34.0	7.1	23.5	15.4	32.6	28.5
>15	22.3	57.1	22.4	30.8	12.5	17.2
NC	6.8	7.1	5.7	9.9	7.0	6.5

NC: information not collected

Antimicrobial resistance: *S. sonnei* showed the highest resistance to trimethoprim-sulfamethoxazole (89.6%; 2,530/2,825) and to tetracycline (more than 80%; 2,278/2,714). Resistance levels for nalidixic acid, chloramphenicol, amoxicillin/clavulanic acid, and ampicillin were 7% (184/2,574), 29.4% (803/2,733), 32.8% (785/2,391), and 52% (1469/2827), respectively (table 2); 14 isolates were resistant to ciprofloxacin, 12 to cefotaxime, 10 to ceftazidime, and 7 to gentamicin.

#### Shigella flexneri

**Figure 3:**
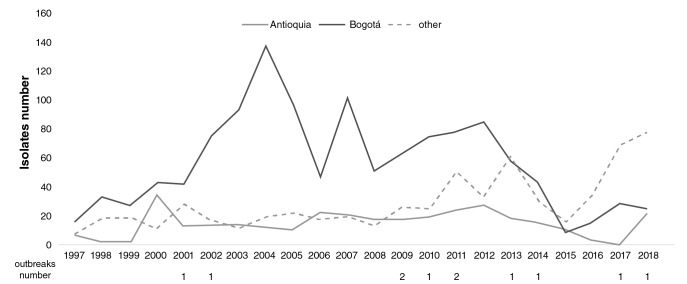
Comparison of the number and distribution of *Shigella flexneri* isolates in Antioquia (grey), Bogotá (black), and others regions (dashed line) in Colombia from 1997 to 2018

A total of 321/2,191 isolates (14.65%) were detected in Antioquia with increases in 2000, 2006, and 2012. Another 627 (28.6%) isolates were recovered in 25 departments representing an increase as of 2016. Eleven outbreaks of *S. flexneri* were notified (figure 3) with 37 related isolates in five departments (Amazonas, Bogotá, Cundinamarca, Meta, and Nariño). The most important outbreak occurred in Cundinamarca in 2001 when 14 isolates were related to food consumed in a school (Figure 3).

Age: The number of *S. flexneri* isolates by age groups was similar. However, almost half of the isolates (48.5%) were recovered in under six-year-old children (table 3).

Antimicrobial resistance: *S. flexneri* highest resistance rates were to tetracycline (94.3% of isolates), ampicillin (83%), chloramphenicol (80.8%), and trimethoprim-sulfamethoxazole (67.8%) (table 2). Less than 10 isolates were resistant to ciprofloxacin, cefotaxime, ceftazidime, and gentamicin.

#### Other species

**Figure 4:**
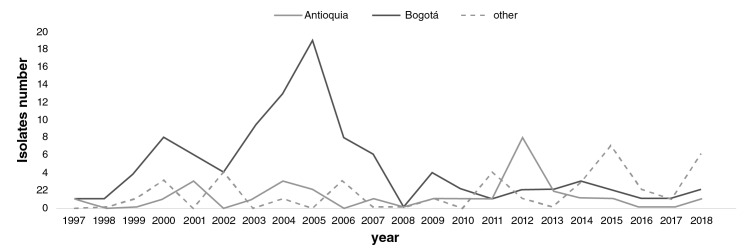
Comparison of the number and distribution of *Shigella* species different to *sonnei* and *flexneri* in Antioquia (black), Bogotá (grey), and other regions (dashed line) in Colombia from 1997 to 2018

Geographical distribution: Out of a total of 164 isolates, 103 were *S. boydii*, 14 were *S. dysenteriae,* and 47 were non-serotypeable *Shigella* isolates. The highest number of *S. boydii* isolates was recovered in 2004 and 2006, the largest number being 42/103 (41%). *Shigella dysenteriae* only appeared in seven of the 22 years surveilled. The highest number of S. *dysenteriae* isolates (6) was recovered in 2018 from Arauca (3), Bogotá (2), and Norte de Santander (figure 4). *Shigella dysenteriae* was not related to outbreaks.

Age: *S. boydii* was recovered mostly in under 14-year-olds (71%) while S. *dysenteriae* was recovered mostly from the >15-year-old population. Non-serotypeable isolates were recovered in patients of all ages (table 3).

Antimicrobial resistance: *S. boydii* exhibited resistance to all evaluated antibiotics except ciprofloxacin. More than 75% of *S. boydii* isolates were resistant to ampicillin and tetracycline and half of these were resistant to trimethoprim-sulfamethoxazole.

*Shigella dysenteriae* was resistant to five of the ten evaluated antibiotics with a lower resistance rate than other species. However, the highest percentage of resistance to nalidixic acid was found in *S. dysenteriae* when compared with other evaluated species (11.1%).

Non-serotypeable isolates showed resistance to seven of the evaluated antibiotics; characteristically, this group had the greatest resistance rate to ciprofloxacin (2.1%) (table 2).

### Antimicrobial resistance evaluated per year and species

#### Beta-lactam antibiotics

Amoxicillin/clavulanic acid: *S. sonnei* resistance began to appear in 2006 when 24.6% of the isolates were resistant; eleven years later, in 2017, this resistance rate reached its highest level of almost 66% (p<0.05). In 2018 the resistance level significantly decreased (11.6%; p<0.05) (Table 4).

**Table 4:** Antimicrobial resistant isolates distribution per year in different *Shigella* species

***Shigella* species**	**n**	**Year/antibiotic/resistance percentage**																					
		**97**	**98**	**99**	**00**	**01**	**02**	**03**	**04**	**05**	**06**	**07**	**08**	**09**	**10**	**11**	**12**	**13**	**14**	**15**	**16**	**17**	**18**
**Tetracycline**																					
*sonnei*	2,714	100	95.0	85.7	95.2	94.9	96.3	97.4	90.8	92.3	92.4	92.0	93.7	82.8	85.6	81.6	86.3	70.0	70.2	70.3	64.2	62.5	41.2
*flexneri*	2,137	96.2	96.2	97.9	95.5	97.6	98.1	98.3	95.8	96.9	97.7	95.7	95.1	90.5	99.1	91.4	91.7	91.2	95.0	90.6	87.0	95.9	83.3
Others	162	100.0	100.0	60.0	100.0	77.8	100.0	90.0	94.1	71.4	100.0	100.0	0.0	33.3	66.7	66.7	54.5	75.0	57.1	50.0	66.7	0.0	37.5
		**Nalidixic acid**																					
*sonnei*	2,574	0.0	0.0	0.0	0.0	0.0	0.6	1.3	1.1	2.4	4.2	1.7	2.1	4.0	3.0	7.0	12.8	18.6	9.4	14.1	6.3	38.1	25.0
*flexneri*	1,882	0.0	0.0	0.0	1.9	1.2	0.9	0.0	2.4	3.1	1.1	2.9	0.0	4.8	3.4	1.3	0.7	2.2	3.4	0.0	20.0	6.0	5.3
Others	146	0.0	0.0	0.0	0.0	0.0	0.0	0.0	5.9	0.0	0.0	0.0	0.0	0.0	66.7	33.3	9.1	0.0	16.7	10.0	0.0	0.0	0.0
		**Amoxicillin/clavulanic acid**																					
*sonnei*	2,391	0.0	0.0	0.0	0.0	0.0	3.2	5.9	3.4	5.4	24.6	17.0	38.0	33.3	59.9	40.4	57.3	43.2	64.7	43.8	49.0	65.9	11.6
*flexneri*	1,755	0.0	0.0	0.0	0.0	0.0	23.4	37.8	22.0	40.9	71.3	66.7	80.0	72.4	76.1	74.3	75.7	78.8	81.7	76.0	62.5	85.5	35.2
Others	130	0.0	0.0	0.0	0.0	0.0	0.0	11.1	0.0	9.5	27.3	28.6	0.0	0.0	33.3	0.0	9.1	0.0	28.6	22.2	0.0	50.0	0.0
		**Ampicillin**																					
*sonnei*	2,827	38.9	35.0	23.8	36.1	35.9	30.7	35.9	37.7	36.9	48.7	41.2	51.8	43.4	71.5	60.1	74.2	64.5	72.7	71.4	64.2	54.7	25.9
*flexneri*	2,166	82.8	88.7	81.3	72.7	62.7	76.4	78.2	86.9	78.3	90.8	80.0	87.7	86.7	87.3	80.4	87.5	86.0	88.5	88.0	83.3	92.8	81.6
Others	160	50.0	100.0	40.0	66.7	66.7	87.5	90.0	88.2	66.7	100.0	100.0	0.0	50.0	66.7	50.0	54.5	25.0	40.0	0.0	33.3	0.0	33.3
		**Trimethoprim-sulfamethoxazole**																					
*sonnei*	2,825	94.4	85.0	76.2	91.6	89.7	92.0	94.8	87.4	94.6	96.6	89.3	95.0	91.9	89.7	75.9	79.0	92.2	94.2	96.9	94.3	87.3	86.6
*flexneri*	2,166	82.8	73.6	85.4	83.0	83.1	80.2	84.9	78.6	78.3	79.3	71.4	69.1	66.7	56.8	58.8	50.0	57.4	56.4	88.0	51.9	48.5	48.8
Others	160	50.0	0.0	40.0	66.7	66.7	25.0	50.0	47.1	66.7	81.8	71.4	0.0	16.7	0.0	50.0	45.5	75.0	40.0	62.5	66.7	100.0	44.4
		**Chloramphenicol**																					
*sonnei*	2,733	11.1	5.0	0.0	1.2	2.6	0.6	9.2	13.7	11.9	16.8	11.4	30.3	20.2	42.5	37.7	60.3	51.4	59.9	54.3	53.8	50.0	21.6
*flexneri*	2,149	69.0	88.7	75.0	64.8	83.1	74.5	84.0	75.6	78.1	87.4	85.5	87.7	85.7	86.3	83.6	85.4	83.1	85.0	75.0	75.9	74.2	76.6
Others	164	50.0	0.0	20.0	0.0	11.1	0.0	20.0	5.9	14.3	18.2	14.3	0.0	0.0	0.0	16.7	9.1	25.0	57.1	40.0	33.3	0.0	11.1

During the first five years of surveillance of *S. flexneri*, no resistance was recorded. Nonetheless, between 2002 and 2005, rates ranged from 22% to 40.9%; a year later, in 2006, they had increased significantly to 71.3% (p<0.05), and then they were stable until 2018 when there was a significant decrease to 35.2% (p<0.05). This was also observed in *S. sonnei* isolates. In the other species, resistance was detected in nine of the 22 years under surveillance and the highest resistance level was detected in 2017 (50%) (Table 4).

Ampicillin: Since surveillance started in 1997 and for 12 years, the resistance in *S. sonnei* has remained above 50%. In 2010, resistance rates increased significantly to 71.5% (p<0.05) and were stable until 2018 when they decreased significantly to 25.9% (p<0.05). In contrast, *S. flexneri* resistance rates to ampicillin remained high in the same period of time ranging from 62.7% to 92.8% (table 4). There were no resistant isolates from other species only in 2008, 2015, and 2017 (table 4).

Cefotaxime and ceftazidime: Resistance to cefotaxime was found in 21 isolates: 12 *S. sonnei*, 8 *S. flexneri,* and 1 *S. boydii* isolate while we detected 16 resistant isolates to ceftazidime: 10 *S. sonnei,* 4 *S. flexneri,* and 2 *S. boydii* isolates.

Tetracycline: From the very first year of *S. sonnei* surveillance, we found resistance levels in more than 90% of the isolates which decreased to 70% as of 2013 reaching the lowest levels in 2018 with 41.2%. *Shigella flexneri* isolates showed more than 90% resistance during 17 of the surveillance years registering the lowest rates (83.3%) in 2018. Resistance rates in other species fluctuated: in some years there were no resistant isolates (2008 and 2017) while resistance levels reached 100% in 1997, 1998, 2000, 2002, 2006, and 2007 (Table 4).

Trimethoprim-sulfamethoxazole: In *S. sonnei*, resistance remained constant during the study period ranging from 75.9% to a maximum of 96.6% in 2006 and 2015, respectively. In *S. flexneri* resistance levels exceeded 65% until 2010 and then decreased to 48.8% in 2018. Regarding other species, resistance rates varied more: in 1998, 2008, and 2010, isolates were completely sensitive while in 2017 resistance rates reached 100% (table 4).

Quinolones and fluoroquinolones: Resistance to nalidixic acid in *S. sonnei* was higher than in other species remaining stable (below 10%) until 2012 with a maximum level in 2017 (38.1%). In 2016 alone, *S. flexneri* resistant isolates exceeded 20% while in other years this level did not exceed 6% (table 4). Ciprofloxacin-resistant isolates amounted to only 24: *S. sonnei* (13), *S. flexneri* (9), and *S. boydii* (1).

Phenicols: The highest resistance rate was detected in *S. flexneri* with more than 70% of isolates during surveillance years. In *S. sonnei* isolates, resistance rates were less than 30% until 2008, when they began to increase until reaching 60.3% in 2012. For other species, resistance levels varied during the surveillance years (table 4).

Aminoglycosides: Gentamicin was evaluated within the aminoglycoside class and we detected 12 resistant isolates: seven were *S. sonnei*, four *S. flexneri,* and one *S. boydii*.

### Resistance profiles

The most frequent resistance profile was detected in 1,001/5,043 (19.8%) isolates and it corresponded to multidrug resistance (resistance to three or more antibiotic classes: TE-C-AMC-AMP-SXT) (Table 5). Resistance rates were similar in *S. flexneri and S. sonnei* (Table 5).

**Table 5:** Antimicrobial resistance profiles distribution in different *Shigella* species

**Resistance profiles**	***Shigella* species**					
	***flexneri*** **n (%)**	***sonnei*** ** n (%)**	***boydii*** **n (%)**	***dysenteriae*** **n (%)**	***Shigella* spp.** **n (%)**	**n isolates**
TE-C-AMC-AMP-SXT	555 (55.4)	442 (44.2)	3 (0.3)	0	1 (0.1)	1,001
TE-SXT	125 (13)	825 (86)	4 (0.4)	1 (0.1)	4 (0.4)	959
TE-AMP-SXT	90 (16.9)	402 (75.6)	33 (6.2)	2 (0.4)	5 (0.9)	532
TE-C-AMP-SXT	410 (77.5)	109 (20.6)	3 (0.6)	1 (0.2)	6 (1.1)	529
TE-C-AMC-AMP	374 (83.5)	73 (16.3)	0	0	1 (0.2)	448
SXT	16 (5.3)	277 (92)	3 (1)	0	5 (1.7)	301
AMP-SXT	27 (17.5)	120 (77.9)	2 (1.3)	1 (0.6)	4 (2.6)	154
TE-C-AMP	141 (91.6)	8 (5.2)	4 (2.6)	0	1 (0.6)	154
TE-C-SXT	128 (87.7)	17 (11.6)	0	0	1 (0.7)	146
TE-AMC-AMP-SXT	30 (21.1)	111 (78.2)	1 (0.7)	0	0	142
Other profiles (n=78)	245 (36.2)	377 (55.7)	40 (5.9)	1 (0.8)	14 (2.1)	677
Total	2,141	2761	93	6	42	5,043

AMC: Amoxicillin/clavulanic acid; TE: Tetracycline; C: Chloramphenicol; NA: Nalidixic acid; CAZ: Ceftazidime; CTX: Cefotaxime; AMP: Ampicillin; SXT: Trimethoprim sulfamethoxasole; CIP: Ciprofloxacin; GM: Gentamicin

Other resistance profiles were specific for each species. The most frequent in *S. flexneri* were TE-C-AMP (91.6%), TE-C-SXT (87.7%), TE-C-AMC-AMP (83.5%), and TE-C-AMP-SXT (77.5%). Interestingly, the main *S. sonnei* resistance profile was to SXT solely (92%; 277/301) followed by resistance to two antibiotics: TE-SXT (86%; 825/959). Furthermore, compared with *S. flexneri,* fewer multidrug-resistant isolates from other species were detected. TE-AMC-AMP-SXT: 78.2%; AMP-SXT: 77.9%, and TE-AMP-SXT: 75.6% were the most frequent resistance profiles (table 5).

## Discussion

Diarrheal diseases continue to cause morbidity and mortality in low- and middle-income countries. Estimates from 195 countries reveal that global diarrhea mortality among individuals older than 5 years has been dominated by *Shigella* spp. (14). Additionally, *Shigella* spp. was the leading cause of diarrheal mortality among people older than 70 years (13,15).

In this report, most of the strains were isolated from under 6-year-old children. *Shigella sonnei* resistance profile included trimethoprim-sulfamethoxazolesolely, which indicates that age could be an important factor in acquiring *Shigella* infection in Colombia. The findings we present were supported by a prospective surveillance study also performed in Colombia to determine the incidence of *Shigella* spp. in acute gastroenteritis that revealed that 23% of the isolates were associated with hospitalization and outpatient care in single infections and coinfections among children between 24 and 59 months of age (16). As described in the Global Enteric Multicenter Study (GEMS), *Shigella* spp. burden increased proportionally with age and became the second most common pathogen identified among 12 to 23 months children and the leading pathogen among 24 to 59 months ones (17).

The dominant species was *S. flexneri* in the first three years (1997 to 1999) and in the last three years of surveillance (2016 to 2018). Since 2000 and for 16 years (2000 to 2015), the dominant species was *S. sonnei* in all the departments under study. This ‘replacement phenomenon’ has been documented in many countries in different regions of the world such as Asia and it supports the emergence of *S. sonnei* replacing *S. flexneri* as the most frequent agent of shigellosis in economically transitional states (18,19).

*Shigella sonnei* and *S. flexneri* were the most common isolates coinciding with reports at global level. For example, reports on the incidence of several *Shigella* species in countries of the Americas evidenced their presence in an urban community in Ecuador and in a Chilean periurban area (20,21). Additionally, the current global epidemiological burden for shigellosis is attributed to these two species: *S. flexneri*, conventionally associated with low- and middle-income countries, and *S. sonnei* with high-income regions (19).

Noticeably, regions that had undergone significant industrialization reported increasing cases of *S. sonnei* compared to low- and middle-income areas where *S. flexneri* levels have remained high (22). It has been suggested that this shifting trend may be due to an improvement of overall nutritional status (23), socioeconomic level, and sanitation conditions (24). It has also been suggested that such shift is mediated by cross-immunity. In less-developed countries, repeated ingestion of *Plesiomonas shigelloides* bacteria through consumption of untreated surface waters may stimulate cross-protection against *S. sonnei* with O antibodies mediating protection (25).

The appearance of *S. flexneri* in our study as the most common species during the last three years of surveillance could be an indicator of a decline in the quality of water supply and good sanitation in Colombian communities. It has been shown that an increase in the provision of clean water and sanitation possibly disrupts *S. flexneri* traditional transmission route (26).

Regarding the other species evaluated in our report, *S. boydii* and *S. dysenteriae*, we observed fewer isolates, which agrees with other worldwide reports (10,17,27), but outbreaks of both species have been reported (28,29).

We recovered *S. dysenteriae* isolates sporadically, one or two strains during the 23 years of surveillance. However, in 2018, six isolates were recovered from three different departments, two of them located in borders (Arauca and Norte de Santander); isolates of these species had not been previously reported in these states. According to previous reports, their presence may indicate the possibility of an epidemic in these or other regions within Colombia (30).

These isolates were evaluated for the presence of Shiga toxin and were negative. Some did not react with any *Shigella* serogroup/serotype-specific antisera. Other studies have found similar results with strains classified as untypable by serotyping in many other countries (31-33).

Regarding the antimicrobial resistance profiles of species under study, we found the highest resistance levels to tetracycline, trimethoprim-sulfamethoxazole, and ampicillin, a trend also observed in Africa, Asia, and other South American countries where ampicillin resistance rates were high for almost all *Shigella* species. In most of the studies in Africa and Asia, *Shigella* serogroups developed resistance to tetracycline, chloramphenicol, and trimethoprim-sulfamethoxazole (34-36).

Additionally, resistance levels to amoxicillin/clavulanic acid reached almost 50% in our study, which was higher than the Latin American mean reported by the Antimicrobial Surveillance Program where 27% of the isolates were resistant to this antibiotic but less frequent than the 71% reported in Europe (35).

Resistance rates to cefotaxime, ceftazidime, gentamicin, and ciprofloxacin were less than 1%. In general, the antimicrobial resistance level for these antibiotics is similar across Latin America where ceftriaxone is active against all *Shigella* spp. and fluoroquinolone-resistant isolates are limited (35,37).

The antimicrobial resistance against specific species revealed that *S. sonnei* had the highest resistance level to trimethoprim-sulfamethoxazole. Indeed, *S. sonnei* resistant isolates were found in all surveillance years. The resistance to amoxicillin/clavulanic acid reached its highest rate in 2017 and abruptly decreased by more than 50% during 2018, which was unexpected considering the behavior of the isolates reported herein.

Only 6% of *S. flexneri* isolates were susceptible to tetracycline, which means that more than 90% of the population is resistant. Likewise, ampicillin and chloramphenicol resistance were higher in *S. flexneri* isolates than in all other evaluated species. A similar situation has been revealed in other parts of the world with high rates of resistance to at least one of the common antibiotics such as ampicillin, tetracycline, and chloramphenicol (38,39).

Contrary to reports of *S. flexneri* resistance to gentamicin, nalidixic acid, and ciprofloxacin (34) in other regions, our resistant isolates did not reach high resistance levels. A similar situation occurred for cephalosporin where resistance was less than 1%, similar to that reported by Lima, *et al*. (40).

The *S. boydii* isolate we analyzed here exhibited resistance to all antibiotics evaluated except for ciprofloxacin. Interestingly, this species had the highest resistance rates to cephalosporin and gentamicin. Regarding tetracycline, ampicillin, and trimethoprim-sulfamethoxazole, the trend was similar to previous reports (34).

We found the highest percentage of resistance to nalidixic acid in *S. dysenteriae* isolates, similar to those in India and Bangladesh where an alarming 82% resistance in this species was reported (41,42). In Egypt, Wasfy, *et al.,* found over 50% resistance levels to chloramphenicol, tetracycline, and ampicillin (43,44). However, compared to other studies (31,45), in ours the isolates had lower levels of resistance to other antibiotics evaluated and were completely sensitive to amoxicillin/clavulanic acid, ceftazidime, cefotaxime, and ciprofloxacin.

An increase in fluoroquinolone resistance in *S. flexneri* and *S. sonnei* isolates in high-income countries has been reported and, although nalidixic acid resistance was common among *S. sonnei* isolates, the trend of fluoroquinolone resistance is slowly increasing (46,47). Oral ciprofloxacin can achieve high concentrations in serum and stool and has activity against *Shigella* isolates. However, ciprofloxacin should not be prescribed for children (48).

In Colombia, quinolones are not generally recommended for shigellosis treatment, which may have contributed to the low levels of resistance observed. Macrolide azithromycin is widely used in children and is recommended as an alternative therapy for the treatment of shigellosis in adults infected with multidrug-resistant isolates (49); however, *in vitro* azithromycin susceptibility testing is not routinely performed by the country’s *Shigella* surveillance system.

Our laboratory-based surveillance of *Shigella* spp. determined that children under five years of age were affected by all of them. These findings should be considered by funders and public health officials to make evidence-based decisions for effective protection and prevention including attainable vaccines. Characteristically, an increased frequency of multidrug resistant S. *flexneri* was observed. Following international epidemiological trends, *S. sonnei* is likely to become more prevalent on a global scale as countries increase their level of development and sanitation. Combating the spread of global antibiotic resistance will be greatly benefited by focusing on *S. sonnei* surveillance while more localized efforts are needed to combat *S. flexneri* resistance.
